# *STAT3* mutations in “gray-zone” cases of T-cell large granular lymphocytic leukemia associated with autoimmune rheumatic diseases

**DOI:** 10.3389/fmed.2022.1000265

**Published:** 2022-08-31

**Authors:** Vadim Gorodetskiy, Yulia Sidorova, Bella Biderman, Natalia Kupryshina, Natalya Ryzhikova, Andrey Sudarikov

**Affiliations:** ^1^Department of Intensive Methods of Therapy, V.A. Nasonova Research Institute of Rheumatology, Moscow, Russia; ^2^Laboratory of Molecular Hematology, National Medical Research Center for Hematology, Moscow, Russia; ^3^Hematopoiesis Immunology Laboratory, Russian Cancer Research Center N.N. Blokhin, Moscow, Russia

**Keywords:** T-cell large granular lymphocytic leukemia, low tumor burden, *STAT3* mutation, next-generation sequencing, autoimmune rheumatic diseases

## Abstract

A persistently increased T-cell large granular lymphocyte (T-LGL) count in the blood of more than 2 × 10^9^/L for at least 6 months is necessary for a reliable diagnosis of T-LGL leukemia. In cases with LGL counts of approximately 0.5–2 × 10^9^/L, a diagnosis of T-LGL leukemia can be made if clonal rearrangement of T-cell receptor (*TCR*) genes is present and if the patient shows typical manifestations of T-LGL leukemia, such as cytopenia, splenomegaly, or concomitant autoimmune disease. However, in cases with LGL counts of less than 0.5 × 10^9^/L, the diagnosis of T-LGL leukemia is questionable (termed as “gray-zone” cases). Although mutations in signal transducer and activator of transcription 3 (*STAT3*) gene are the molecular hallmark of T-LGL leukemia, their diagnostic value in the “gray-zone” cases of T-LGL leukemia has not been evaluated – our study has been aimed to examine the prevalence of *STAT3* mutations in these cases. Herein, we describe 25 patients with autoimmune rheumatic diseases, neutropenia, clonal rearrangement of *TCR* genes, and circulating LGL count of less than 0.5 × 10^9^/L. Splenomegaly was observed in 19 (76%) patients. Mutations in the *STAT3* were detected in 56% of patients using next-generation sequencing. Importantly, in 3 patients, no involvement of the blood and bone marrow by malignant LGLs was noted, but examination of splenic tissue revealed infiltration by clonal cytotoxic T-lymphocytes within the red pulp, with greater prominence in the cords. We suggest using the term “splenic variant of T-LGL leukemia” for such cases.

## Introduction

Large granular lymphocytic (LGL) leukemia is characterized by the expansion of lymphocytes with abundant cytoplasm containing variably sized azurophilic granules and a reniform or round nucleus with mature chromatin (1). Approximately 85% of LGL leukemia patients exhibit T-cells with a mature post-thymic phenotype (1). T-cell LGL (T-LGL) leukemia has an indolent clinical course and involves the peripheral blood, bone marrow, and spleen (2). A typical manifestation of T-LGL leukemia includes neutropenia, splenomegaly, and LGL lymphocytosis, which are detected in up to 84%, 50%, and 52% of patients, respectively (3). Moreover, up to 40% of patients with T-LGL leukemia have concomitant autoimmune disorders, of which rheumatoid arthritis (RA) is the most common (1, 4, 5).

Historically, a definitive diagnosis of LGL leukemia is made if the LGL count in the peripheral blood is more than 2 × 10^9^/L (normal range, < 0.3 × 10^9^/L) over a period of 6 months, with no clearly identified cause (3, 6). Although this definition of T-LGL leukemia remained unchanged in the 2016 revision of the World Health Organization (WHO) classification of tumors of hematopoietic and lymphoid tissues, cases wherein the LGL count is less than 2 × 10^9^/L may be suggestive of this diagnosis (2). While a lower threshold of the absolute LGL count in the peripheral blood for T-LGL leukemia has not been defined by the WHO, the threshold of 0.5 × 10^9^/L is now generally accepted (5, 7). In cases with LGL counts of approximately 0.5–2 × 10^9^/L, a diagnosis of T-LGL leukemia can be made if the LGLs are clonal and if the patient shows typical manifestations of T-LGL leukemia, such as cytopenia, splenomegaly, or concomitant autoimmune disorder (5, 7–10). Infiltration of bone marrow by clonal T-LGLs and/or characteristic histopathological findings of LGL leukemia in the bone marrow with typical manifestations of T-LGL leukemia also confirm the diagnosis of T-LGL leukemia (8, 11, 12). However, cases with circulating LGL counts of less than 0.4–0.5 × 10^9^/L (termed as “gray-zone” cases) present a specific diagnostic challenge because of the difficulty in making a differential diagnosis with reactive T-LGL proliferation.

Although T-cell clonality is necessary to distinguish T-LGL leukemia from reactive T-LGL proliferation, clonality does not equate to malignancy and is not enough to classify T-LGL proliferation as leukemia (13, 14).

Constitutive activation of the signal transducer and activator of transcription 3 (STAT3) signaling pathway is a molecular hallmark of T-LGL leukemia (5). Activating point mutations in the *STAT3* gene are found in up to 72% of T-LGL leukemia patients (15–18). Thus, detection of *STAT3* mutations may serve as a valid reason for classifying the condition in diagnostically challenging cases as T-cell LGL leukemia (15). However, their prevalence in “gray-zone” cases has not been evaluated.

In the present study, *STAT3* gene mutations were evaluated using next-generation sequencing (NGS) in a cohort of 25 patients with autoimmune rheumatic diseases (ARDs), clinical presentation typical for T-LGL leukemia, clonal rearrangement of the T-cell receptor (*TCR*) gene, and absolute circulating LGL count of less than 0.5 × 10^9^/L.

## Patients and methods

**FIGURE 1:**
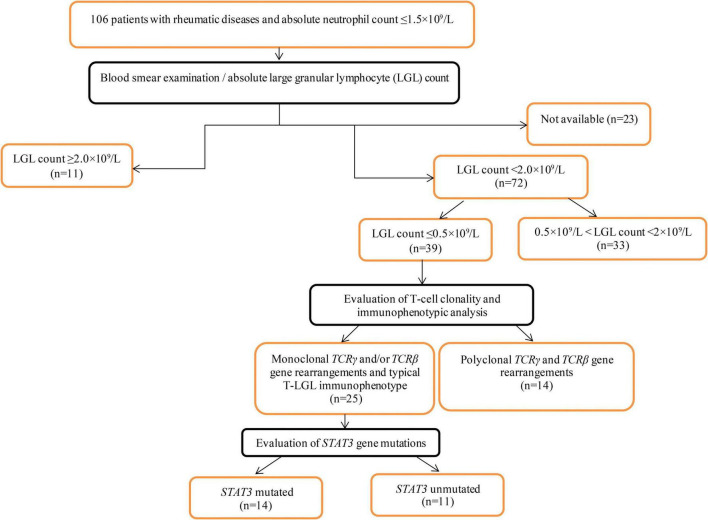
A flowchart of the patient selection process.

The inclusion criteria were as follows: patients aged > 18 years with diagnosed ARD and absolute neutrophil counts of < 1.5 × 10^9^/L. From 2008 to 2021, 106 patients admitted to V.A. Nasonova Research Institute of Rheumatology met the inclusion criteria and were included in the primary analysis. A flowchart of the patient selection process is shown in Figure 1.

Peripheral blood smears were available for 83 patients. Manual differential tests were performed using Wright-Giemsa-stained peripheral blood smears to quantify the LGL percentage and calculate the absolute LGL counts. The circulating LGL count was ≤ 0.5 × 10^9^/L in 39 patients. In 25 of the 39 patients, T-cell clonality testing revealed clonal populations. These 25 patients were included in the final analysis.

The clinical data of patients were collected, including age of onset of neutropenia or splenomegaly, sex, rheumatologic diagnosis, and bone marrow differential counts. Splenic tissue specimens (from 5 patients) and bone marrow trephine biopsies (from 15 patients) had previously been fixed in 10% formalin, routinely processed, embedded in paraffin, and stained with hematoxylin and eosin.

Screening for *STAT3* mutations was performed on samples in which a monoclonal *TCR* gene rearrangement pattern was detectable. For samples with a polyclonal *TCR* gene rearrangement pattern, *STAT3* mutation screening was conducted if a monoclonal *TCR* gene rearrangement pattern was detected in other tissue samples from the same patient. For example, if a monoclonal *TCR* gene rearrangement was detected in the bone marrow but was absent in the blood sample, screening for *STAT3* mutations was performed in both the bone marrow and blood samples.

### Ethics statement

This study was approved by the Ethics Committee of the V.A. Nasonova Research Institute of Rheumatology (protocol #10 on the 20-01-2022). All patients gave written consent for collection, analysis of specimens and for publication of their data as results of the study.

### Evaluation of T-cell clonality

T-cell clonality was examined using genomic DNA extracted from blood (25 patients), bone marrow (16 patients), and spleen tissue (5 patients) samples. T-cell clonality was evaluated based on the rearrangements of *TCR*-γ (Vγ–Jγ) and *TCR*-β (Vβ–Jβ, Dβ–Jβ) in all patients. T-cell clonality assays were performed according to the BIOMED-2 standardized protocol (19). Polymerase chain reaction (PCR) amplification was conducted using an automated DNA Engine Thermal Cycler (Bio-Rad, Hercules, United States), and fragments were detected using an ABI PRISM 3130 Genetic Analyzer (Applied Biosystems, Foster City, CA, United States). The data were analyzed using GeneMapper software version 4.0 (Applied Biosystems).

Detection of a weak clonal signal in the context of polyclonal signals was interpreted as ambiguous clonality. False positive results (pseudo-monoclonality) were excluded by identical dominant PCR products in repeated PCR analyses of the same sample and/or identical dominant PCR products in different test samples (blood, bone marrow, or spleen samples) from the same patient.

### Immunophenotypic analysis

Immunohistochemical studies of the spleen (5 patients) and bone marrow (15 patients) were conducted using the formalin-fixed paraffin-embedded tissue. The following antibodies were used at dilutions suggested by the manufacturers: CD3 (polyclonal, Dako, Carpinteria, CA, United States); CD4 (clone 4B12, Dako); CD8 (clone C8/144B, Dako); CD16 (clone 2H7, Novocastra Laboratories, Newcastle upon Tyne, United Kingdom); CD20 (clone L26, Dako); granzyme B (clone GrB-7, Dako); T-cell restricted intracellular antigen 1 (TIA-1) (clone 2G9, Immunotech, France); TCR-β F1 (clone 8A3, Thermo Scientific, Waltham, MA, United States); and TCR-γ (clone γ3.20, Thermo Scientific). After dewaxing and heat-induced antigen retrieval, immunostaining was performed using an Autostainer Link 48 (Dako, Denmark) according to the manufacturer’s instructions. All immunostained samples were counter-stained with hematoxylin.

### Evaluation of *STAT3* mutations

*STAT3* mutations were examined using genomic DNA extracted from specimens of peripheral blood (24 patients), bone marrow (15 patients), and the spleen (2 patients).

Mutations in exons 19–21 of the *STAT3* gene were identified by NGS. Appropriate DNA regions were amplified using primers for exons 19–20 (product length 502 bp) and exon 21 (product length 522 bp), as described previously (15).

Amplified DNA fragments were converted to sequencing libraries using Nextera XT DNA Library Prep and Nextera XT Index Kit v2 (Illumina, United States) according to the manufacturer’s instructions. Nucleotide sequences were analyzed on a MiSeq sequencer (Illumina) using MiSeq Reagents Kit v2 for 300 cycles (Illumina). Raw data filtering, removal of accessory sequences, mapping of reads, and searching for variants were performed using the SAMtools (20), BWA (21), Trimmomatic (22), and VarDict (23). Usually, 2,000–5,000 reads were obtained for each target, and the cutoff for reporting variants was set to 0.5%. Discovered variants were annotated with ANNOVAR (24) utility using snp138 (25), refGene (26), ClinVar (27), and COSMIC (28) open databases. In two samples, *STAT3* mutations were assessed by real-time allele-specific PCR assay for the most common substitutions: Y640F, D661Y, N647I, D661V, D661H, and D661N.

### Statistical analysis

Descriptive statistics are presented as numbers and percentages for categorical data and as medians and ranges for continuous data. A two-sample test for equality of proportions with continuity correction was used for statistical analysis. A *p*-value of < 0.05 was considered statistically significant.

## Results

The clinical, laboratory, and pathologic characteristics of the 25 patients included in the final analysis are shown in Table 1. According to the inclusion criteria, all patients had ARD, neutropenia counts of ≤ 1.5 × 10^9^/L (median 0.240 × 10^9^/L, range, 0.016–1.428 × 10^9^/L), LGL counts of ≤ 0.5 × 10^9^/L in the peripheral blood (median 0.381 × 10^9^/L, range, 0.028–0.499 × 10^9^/L), and a clonal T-cell population.

**TABLE 1:** Characteristics of 25 patients with rheumatic diseases, clonal T-LGL proliferations, and LGL counts of ≤ 0.5 × 10^9^/L in blood.

Patient no./Sex/Age (y)*	RD	Splenomegaly	Absolute neutrophil count (×10^9^/L)	Absolute lymphocyte count (×10^9^/L)	Absolute LGL count (×10^9^/L)	Percentage of lymphocytes in BM aspirate	Interstitial/Intra sinusoidal distribution of cytotoxic T cells in BM	Samples for testing T-cell clonality and STAT3 mutation	T-cell clonality		Variants of *STAT3* mutation (VAF)
										
									γ	β	
1/F/65	RA	+	0.120	0.880	0.275	39.6	+	PB	Mono	Mono	S614R (2%)
2/F/59	RA	+	0.208	0.728	0.212	21.2	ND	PB	Mono	Mono	–
3/M/49	RA	+	1.078	0.814	0.352	8.1	+	PB	Poly	Mono (ambiguous)	–
								BM	Poly	Mono (ambiguous)	–
4/F/46	RA	+	0.084	0.876	0.276	10.4	–	PB	Poly	Mono	–
								Spleen	Poly	Mono	ND
5/F/67	RA	+	0.900	1.200	0.425	17.0	ND	PB	Mono	Mono	Y640F (9.2%)
6/F/51	RA and sSS	+	0.610	0.240	0.095	18.2	–	PB	Mono	Mono	–
								BM	Mono	Mono	–
7/F/55	RA	–	1.323	1.242	0.486	22.4	ND	PB	Poly	Mono	–
								BM	Poly	Mono	Y640F (1.7%)
8/F/35	RA	+	1.092	1.222	0.381	25.6	+	PB	Poly	Mono (ambiguous)	–
								BM	Poly	Mono (ambiguous)	–
9/M/39	RA	+	0.126	0.490	0.196	22.6	+	PB	Poly	Poly	–
								BM	Poly	Poly	–
								Spleen	Mono	ND	ND
10/M/71	RA	+	0.044	1.342	0.440	17.0	ND	PB	Poly	Poly	–
								BM	Mono	Mono	–
11/F/58	RA	+	0.112	0.476	0.063	18.4	–	PB	Poly	Poly	–
								BM	Poly	Poly	S614R (0.8%)
								Spleen	Mono	Poly	S614R (9.2%)
12/F/64	RA	+	0.160	1.344	0.336	25.8	+	PB	Poly	Mono	D661Y (6.7%)
								BM	Mono	Mono	D661Y (5.9%)
13/F/67	RA	+	1.428	1.148	0.126	8.4	ND	PB	Poly	Mono (ambiguous)	Y640F (1.2%)
								BM	Mono (ambiguous)	Mono	Y640F (0.8%)
14/F/60	RA and sSS	+	0.812	1.653	0.479	9.0	+	PB	Mono	Mono	N647I (8%)
15/F/43	RA	+	0.016	1.376	0.448	22.8	+	PB	Poly	Mono	–
16/F/53	RA and sSS	–	1.332	1.776	0.499	41.6	+	PB	Poly	Mono	Y640F (3.4%)
								BM	Poly	Mono	Y640F (5.4%)
17/M/63	RA	–	0.408	1.258	0.434	ND	ND	PB	Mono (ambiguous)	Mono	Y657_K658insY (2.6%)
18/M/60	RA	+	0.154	0.756	0.455	10.0	–	PB	Poly	Mono (ambiguous)	D661Y (2.8%)
								Spleen	Mono	Mono	D661Y (#)
19/F/61	RA and sSS	+	0.196	0.420	0.028	16.8	ND	PB	Mono	Poly	D661Y (3.7%)
								BM	Mono	Mono	D661Y (4.7%)
20/F/69	RA	+	0.240	0.460	0.320	5.2	–	PB	Poly	Poly	–
								BM	Poly	ND	ND
								Spleen	Mono	Mono	ND
21/F/61	RA	+	0.022	1.782	0.484	17.8	ND	PB	Poly	Poly	–
								BM	Poly	Mono (ambiguous)	–
22/M/52	RA	+	0.055	0.77	0.297	20.2	+	PB	Poly	Poly	ND
								BM	Mono	Mono	D661Y (#)
23/F/56	SLE and sSS	–	1.085	1.240	0.413	17.2	+	PB	Poly	Mono	K658R (1.2%)
								BM	Poly	Mono	E616G (0.6%); K658R (< 0.5%)
24/F/50	SLE and sSS	–	1.272	0.960	0.456	ND	ND	PB	Poly	Mono	–
25/F/51	pSS	–	1.029	0.735	0.441	6.6	ND	PB	Poly	Mono	N664T (4.5%); D661Y (4.3%)
								BM	Mono	Mono	N664T (1%); D661Y (0.9%)

Symbol * at the time of detection neutropenia or splenomegaly; y, years; RD, rheumatic disease; RA, rheumatoid arthritis; SLE, systemic lupus erythematosus; pSS, primary Sjogren’s syndrome; sSS, secondary Sjogren’s syndrome; BM, bone marrow; PB, peripheral blood; +, positive/present; –, negative/absent; ND, no data; LGLs, large granular lymphocytes; TCR, T cell receptor; Mono, monoclonal TCR gene rearrangement; Poly, polyclonal TCR gene rearrangement; *STAT3*, signal transducer and activator of transcription 3 gene; VAF, variant allele frequency. #, mutation was detected by allele-specific real-time polymerase chain reaction assay.

Overall, 22 patients had RA, 2 had systemic lupus erythematosus (SLE), and 1 had primary Sjogren’s syndrome (SS). Six patients had SS in combination with other rheumatic diseases (secondary SS): 4 with RA and 2 with SLE.

The median age of the patients at the time of neutropenia detection and/or splenomegaly was 58 years (range, 35–71 years). The female-to-male ratio was 3.2:1. Splenomegaly was observed in 19 (76%) patients, among whom 5 underwent splenectomy for diagnostic or therapeutic purposes. The absolute lymphocyte counts in the blood ranged from 0.24 to 1.782 × 10^9^/L, with a median of 0.96 × 10^9^/L.

Bone marrow aspirate differential counts were available for 23 patients. In all cases, there were no signs of myelodysplasia. The proportion of lymphocytes in the bone marrow was between 5.2% and 41.6% (median 17.8%) of all nucleated cells, but only 4 (17%) patients had bone marrow lymphocytosis, defined as exceeding the upper limit of lymphocytes of > 23.8% of nucleated bone marrow cells. Interstitial/intrasinusoidal distribution of cytotoxic T-cells in the bone marrow was detected in 10 (67%) of the 15 patients examined.

Examination of splenic tissue revealed lymphoid infiltration of both splenic cords and sinusoids within the red pulp, with greater prominence in the cords. The white pulp was preserved and contained prominent germinal centers in 3 patients and was atrophied in 2 patients. Immunohistochemical staining revealed that the lymphocytes infiltrating the red pulp were CD3^+^/TIA1^+^ in all patients and CD16^+^ in 4 patients. Beta-F1 and TCR-γ positivity was observed in 3 and 1 patient, respectively. In another patient, approximately one-third of the T-cells expressed Beta-F1, and another third of the T cells expressed TCR-γ. In addition, approximately one-third of the T-cell population did not express TCR. Lymphocytes were CD8^+^/CD4^–^ in three TCRαβ^+^ cases and CD8^–^/CD4^–^ in the remaining cases.

Clonal (or ambiguous clonal) rearrangement of *TCR* genes in the peripheral blood was detected in 19 of the 25 patients: *TCR*-γ gene in 7 (28%) of the 25 patients and *TCR*-β gene in 18 (72%) of the 25 patients. Out of 16 bone marrow samples examined, clonal (or ambiguous clonal) rearrangement of *TCR* genes was detected in 13 cases (in 3 of these patients, it was not detected in the blood): *TCR*-γ gene in 7 (44%) of 16 patients and *TCR*-β gene in 13 (87%) of 15 patients (in one case this study was not performed). Clonal *TCR*-β gene rearrangements were detected more often than *TCR*-γ gene rearrangements both in the peripheral blood (72 vs. 28%, *p* = 0.004678) and bone marrow samples (87 vs. 44%, *p* = 0.03399). Clonal rearrangements of *TCR* genes in the spleen samples were detected in all 5 patients examined: *TCR*-γ gene in 4 of 5 patients and *TCR*-β gene in 3 of 4 patients (in one case this study was not performed). Overall, a clonal (or ambiguous clonal) rearrangement of *TCR* genes was detected in the blood and/or bone marrow samples in 22 (88%) of the 25 patients. In the 3 remaining patients, it was found only in the spleen samples.

Point mutations in the *STAT3* gene were identified in 14 (56%) of the 25 patients examined, including D661Y (5 patients), Y640F (4 patients), S614R (2 patients) and N647I, Y657_K658insY, K658R, N664T, and E616G in one patient each. Double mutation in the *STAT3* gene was observed in 2 patients. *STAT3* mutations were detected in 11 (46%) of 24 blood samples, 9 (60%) of 15 bone marrow samples, and in both the spleen samples (100%). In one bone marrow sample, *STAT3* mutation was detected in the absence of a clonal rearrangement of the *TCR* genes.

## Discussion

Clonal proliferation of T-LGLs has been detected in various clinical conditions, including autoimmune disorders, myeloid or lymphoid clonal hematologic malignancies, pure red cell aplasia, aplastic anemia, and paroxysmal nocturnal hemoglobinuria (29–33). In addition, small clonal proliferations of T-LGLs have been observed after allogeneic stem cell transplantation (34, 35) and solid organ transplantation (36–38) as well as in patients with HIV (39) or cytomegalovirus infection (40). It is likely that some of these cases may represent an intensive immune response to antigen exposure more than would be expected for physiological T-cell clones, rather than true T-LGL leukemia. Although the etiology and pathogenesis of T-LGL leukemia remains unclear, the triggering event underlying T-LGL leukemia is believed to be related to chronic antigenic stimulation initially leading to a poly- or oligoclonal expansion of LGLs (41, 42). The proliferation of LGLs is supported by several cytokines, including interleukin (IL)-6, IL-12, and IL-15 (43–47). The activating point mutations of *STAT3* in these antigen-driven LGLs can cause aberrant STAT3 signaling and, in combination with other factors, ultimately lead to the monoclonal expansion of LGLs (42, 48).

Monoclonal expansions of CD8^+^ T-lymphocytes have also been detected in healthy individuals (49–52). Bigouret et al. suggested that T-LGL clones exist in a significant number of healthy individuals and clinical symptoms, such as neutropenia and autoimmune diseases, can occur when T-LGL clone encounters an antigen and begins producing large amounts of effector molecules (49).

The diagnosis of T-LGL leukemia in cases with small clonal populations in blood has been debated. The term “T-cell *clonopathy* of undetermined significance” was coined to emphasize the indolent clinical course of T-LGL leukemia in many patients and to eliminate the stigma associated with the term “leukemia.” The term “T-cell *clonopathy* of undetermined significance” has been used regardless of the peripheral blood LGL count, clinical symptoms, and hematological abnormalities observed in patients (53, 54). Subsequently, the term “T-cell *clone* of undetermined significance” (TCUS) was proposed for patients with a small clonal population of T-LGL and no diagnostic features of T-LGL leukemia (51, 55). However, the diagnostic threshold of circulating LGLs to distinguish T-LGL leukemia from TCUS has not been defined, and the researchers proposed a threshold range from 500 cells/μL (51) to 2,000 cells/μL (55). In addition, the immunophenotypic signature of TCUS closely resembles that of T-LGL leukemia (51), and the immunohistochemical pattern of bone marrow in TCUS has not been studied yet.

Activating point mutations in *STAT3* are not pathognomonic for T-LGL leukemia and can be detected in various hematological and non-hematological malignancies (56). In addition, Kim et al. recently found somatic *STAT3* mutations in CD8^+^ T-cells of healthy blood donors carrying the human T-cell leukemia virus type 2 (57). However, in an appropriate clinical context, *STAT3* mutations are a molecular marker that is highly specific for T-LGL leukemia (15–18, 56). Nevertheless, the significance of *STAT3* mutations in distinguishing T-LGL leukemia from TCUS is not clear.

Similar to the *MYD88*L265P mutation, which alone is not an absolute diagnostic marker for Waldenstrom’s macroglobulinemia and is found in 50–80% of patients with IgM monoclonal gammopathy of uncertain significance (58), *STAT3* mutations alone are unlikely to allow a reliable diagnosis of T-LGL leukemia when clonal T-LGL proliferation is found.

Monoclonal B-cell lymphocytosis (MBL) is a hematological condition characterized by the presence of a monoclonal population of B-lymphocytes with a count of less than 5 × 10^9^/L in the peripheral blood and without other features of a B-cell lymphoproliferative disorder, such as cytopenia, organomegaly, lymphadenopathy, or extramedullary involvement (59). The genomic aberrations characteristic of chronic lymphocytic leukemia (CLL) can be found in cases of MBL with an immunophenotype similar to that observed in CLL (60). Drawing an analogy between TCUS/T-LGL leukemia and MBL/CLL, it seems logical that the distinction between T-LGL leukemia and TCUS should be based on the blood LGL count and/or the presence of signs of T-LGL leukemia, such as cytopenia and/or splenomegaly.

Although all patients in our cohort had absolute LGL counts in the peripheral blood below the currently accepted threshold of 0.5 × 10^9^/L, the typical manifestations of T-LGL leukemia, including neutropenia and splenomegaly, allowed us to classify these cases as T-LGL leukemia rather than TCUS.

Patients with *STAT3* mutations develop RA and neutropenia more often than patients without these mutations (15, 16, 61). This likely explains the higher frequency of *STAT3* mutations in our cohort than in T-LGL leukemia patients in two recently reported large studies: 56% vs. 36%–40% (61, 62).

The majority (88%) of patients in our cohort had RA. Manifestations of T-LGL leukemia in the setting of RA are sometimes clinically indistinguishable from Felty’s syndrome (FS), a rare subset of seropositive RA complicated by neutropenia and often splenomegaly. The detection of a clonal rearrangement of TCR genes and/or an expanded T-LGL population (≥ 2 × 10^9^/L) supports the diagnosis of T-LGL leukemia but not of FS (63–66). As reported previously, PCR-based *TCR* gene rearrangement analysis according to the BIOMED-2 protocol followed by GeneScan analysis made the detection of clonal T-cell populations possible with a sensitivity of 5% (19, 67). Due to the low clonal expansion of T-LGLs in “gray-zone” cases, detection of a clonal peak may be impossible or questionable, making diagnosis much more difficult. Immunohistochemical examination of bone marrow biopsies is recommended in “gray-zone” T-LGL leukemia cases (7), but due to the expansion of reactive cytotoxic T-lymphocytes in patients with RA, this study cannot reliably distinguish T-LGL leukemia in the setting of RA from FS (64, 66).

Because *TCR*-γ gene rearrangement occurs in both αβTCR^+^ and γδTCR^+^ T-cells, it is the best target for analysis of T-cell clonality (19, 67). On the other hand, the *TCR*-β gene has more expansive combinatorial diversity in contrast to the *TCR*-γ gene, making the *TCR*-β gene the preferred target for identifying a true clonal process in αβTCR^+^ proliferation. In our patient cohort, a clonal rearrangement of the *TCR*-β gene was detected significantly more often than monoclonal rearrangements of the *TCR*-γ gene both in the peripheral blood and bone marrow. According to the BIOMED-2 group recommendations (68, 69), *TCR*-γ and *TCR*-β gene rearrangements were analyzed simultaneously in our work. Blood and bone marrow examination provided complementary information and allowed the detection of T-cell clonality in 22 (88%) of the 25 patients.

Three patients in our cohort presented a particular diagnostic challenge. These patients had seropositive RA, severe neutropenia, and massive splenomegaly, which, in the absence of evidence for clonal proliferation of LGLs in the blood and bone marrow, allowed an initial misdiagnosis of FS. Morphological examination of spleen samples showed infiltration of cords and sinusoids of the red pulp by cytotoxic T-lymphocytes with more marked involvement within the splenic cords, which is characteristic of spleen involvement by T-LGL leukemia (70). A monoclonal TCR gene rearrangement was detected in the spleen in all the 3 patients, and a *STAT3* mutation was present in 1 of them. These patients presented an unusual variant of T-LGL leukemia, in which tumor cells are localized in the spleen, while very few or no tumor cells in the blood and bone marrow prevented the diagnosis of T-LGL leukemia. Because the term “leukemia” is used when there is extensive peripheral blood and bone marrow involvement, we suggest the term “splenic variant T-LGL leukemia” for such cases.

In conclusion, we have described a cohort of patients with various ARDs and expansion of T-LGLs of less than 0.5 × 10^9^/L in the peripheral blood. Such cases present a diagnostic challenge because of the low clone size and require both *TCR*-γ and *TCR*-β gene rearrangement analysis in the blood and bone marrow. Although cases with a very small clonal population of T-LGLs remain the subject of terminological debate (T-LGL leukemia versus TCUS), clinical manifestations such as neutropenia and splenomegaly have allowed us to classify these cases as T-LGL leukemia.

*STAT3* mutations were detected by NGS in peripheral blood, bone marrow, and spleen in a total of 56% of patients in our cohort, providing an argument for considering these cases as atypical aleukemic presentation of T-LGL leukemia. In diagnostically unclear cases of neutropenia and/or splenomegaly in patients with ARDs, the detection of *STAT3* mutations by NGS can indicate the diagnosis of T-LGL leukemia.

## Data availability statement

The data presented in this study are deposited in the SRA repository, accession number: PRJNA867539.

## Ethics statement

The studies involving human participants were reviewed and approved by the V.A. Nasonova Research Institute of Rheumatology (protocol #10 on the 20-01-2022). The patients/participants provided their written informed consent to participate in this study.

## Author contributions

VG: data collection, analysis and interpretation, literature search, study design, and writing – review and editing the manuscript. YS, BB, NK, NR, and AS: data collection, analysis and interpretation, literature search, editing, and reviewing of the manuscript. All authors contributed to the manuscript and approved the submitted version.
